# Male Courtship Pheromones Induce Cloacal Gaping in Female Newts (Salamandridae)

**DOI:** 10.1371/journal.pone.0144985

**Published:** 2016-01-15

**Authors:** Sunita Janssenswillen, Franky Bossuyt

**Affiliations:** Amphibian Evolution Lab, Biology Department, Vrije Universiteit, Brussel, Belgium; CNRS, FRANCE

## Abstract

Pheromones are an important component of sexual communication in courting salamanders, but the number of species in which their use has been demonstrated with behavioral evidence remains limited. Here we developed a behavioral assay for demonstrating courtship pheromone use in the aquatically courting Iberian ribbed newt *Pleurodeles waltl*. By performing an in-depth study of the courtship behavior, we show that females invariably open their cloaca (cloacal gaping) before engaging in pinwheel behavior, the circling movement that is the prelude to spermatophore uptake. In contrast, cloacal gaping was not observed in failed courtships, where females escaped or displayed thanatosis. Since gaping mainly occurred during male amplexus and cloacal imposition, which is the obvious period of pheromone transfer, we next investigated whether male courtship water (i.e., water holding courtship pheromones) alone was able to induce this reaction in females. These tests showed that courtship water induced cloacal gaping significantly more than water, even in the absence of a male. Cloacal gaping thus provides a simple and robust test for demonstrating courtship pheromone use in the Iberian ribbed newt. Since opening the cloaca is an essential prerequisite for spermatophore pick-up in all internally fertilizing salamanders, we hypothesize that variations on this assay will also be useful in several other species.

## Introduction

Courtship pheromones are chemical signals that elicit a specific reaction in conspecifics of the opposite gender during courtship [1]. Males of internally fertilizing salamanders use multiple courtship pheromones to enhance female responses during their courtship rituals [2–6], and researchers have developed various behavioral tests in trying to demonstrate effective use of these molecules. In the terrestrially courting lungless salamanders (Plethodontidae), courtship behavior among species is remarkably homogeneous [3], and consists of a tail-straddling walk in which the female holds her chin on the male’s tail base, while both move forward until the spermatophore transfer has occurred. For this family, a behavioral test has been designed in which the pheromone-producing mental glands of the male were removed and courtship duration of couples was compared with and without the application of pheromones [7,8]. In contrast to plethodontids, salamandrids are mainly aquatically courting, have no mental gland, and are characterized by a wide diversity in courtship strategies [3,9,10]. Pheromone communication in Salamandridae, which possess dorsal glands that open into the cloaca, has mostly been studied using two-choice tests. Such tests, which give an animal the choice between a chemical and an empty source, have been applied in several variations, such as two floating sponges [2,11–14], linear olfactometers [15], Y-mazes [16–19], and two-choice aquaria [20]. However, these tests are choice-based and do not necessarily allow the natural courtship responses of the animals. Recently, the development of a two-female test in alpine newts (*Ichthyosaura alpestris*) [21] and palmate newts (*Lissotriton helveticus*) [6] showed to be effective for inducing the natural female responses under influence of courtship pheromones. In nature, males of these species typically tail-fan pheromones from their cloaca towards the female, and both sexes have limited contact during the entire courtship display [9]. The female responds to pheromones by showing following behavior and tail-touching [21], which are the prelude for a successful spermatophore transfer with a male [22,23]. In the two-female test, the animals display identical behavioral responses when exposed to courtship water (i.e. water in which a male has been courting, thus containing pheromones) in the absence of a male. By exposing the females to isolated courtship water molecules, it was possible to identify the courtship pheromones by scoring the typical following and tail-touching responses [6].

While most species in the nested clade of Modern Eurasian salamandrids court with limited contact, several other salamanders use amplexus (i.e. the male grasps the female with his limbs) during courtship, rendering the quantification of following behavior less appropriate as a pheromone test for these animals. In aiming to construct a bioassay that might be useful for identifying courtship pheromones across salamandrids, we here investigated whether pheromones can induce a common female response that is independent of species-specific courtship behavior. We used *Pleurodeles waltl*, a newt that displays a sequence of several courtship behaviors (tail-fanning, ventral amplexus, head-to-head amplexus, pinwheel sperm transfer) [24,25] to search for such a response. Our observations and subsequent tests show that females of this species display cloacal gaping (i.e. they open their cloaca) as a response to male pheromones, and that quantification of this behavior allows demonstrating the use of chemical communication during courtship.

## Materials and Methods

### Animal housing and husbandry

A population of 31 animals (10 males and 21 females) was bought from a hobby breeder, and was housed in 3 large containers (L: 78 cm, W: 60 cm, H: 50 cm) with aged tap water (water depth: 25cm; water temperature: 18°C). Genders were separately housed and distributed over the 3 containers. To induce their courtship mood, the animals were thoroughly fed and the water depth was increased with 15 cm, and 25% of the water was refreshed daily. The population was recognized as ready-to-mate by the development of nuptial pads in males and the presence of swollen bellies in females [26]. Animals were subjected to one set-up every other day. The animals are currently kept in the lab under the same conditions, where they will be used for future pheromone research.

### Ethical note

To approximate environmental conditions, housing containers were placed in front of large windows that allowed natural sunlight and natural day-night rhythms. Additionally, the containers were supplied with water plants, stones and wooden hiding places for maximum comfort of the animals. Animals were disturbed as little as possible by handling them only once per other day with clean wetted gloves. To minimize stress during the tests, carton fences were placed around the transparent test containers. All experiments complied with EU and Belgian regulations concerning animal welfare. All experiments were approved by the Ethical Committee for Animal Experiments of the Vrije Universiteit Brussel (project number ECAE14-220-35), and performed accordingly.

### Analysis of courtship behavior

To observe the courtship of *P*. *waltl* in detail, one male and one female were placed in a plastic container (25x16x14 cm) filled with 2L of aged tap water. 42 combinations were observed from top, side and bottom view. Each female was observed twice with two different males. Eleven randomly picked couples were timed during the entire courtship sequence.

### Cloacal gaping test

Two females were placed in a plastic container (25x16x14 cm) filled with 2L of courtship water (CW; see below) for 60 minutes (this duration was chosen based on the above observations of courtship behavior; see Results and Discussion). As a negative control, the same test was performed in 2L of aged tap water. Every five minutes, we filmed each female’s cloaca from the bottom of the container without interrupting the test. Three pictures per video were taken, and the average of the three pictures per female and per event (every five minutes) was used to calculate the cloacal gaping response. The latter was calculated as Relative Cloacal Gaping (RCG), i.e., the width of the cloaca (measured orthogonal to the centre of the cloacal length) divided by the width of the tail base (measured at the hind limb initiation region). If the cloacal opening was invisible, the RCG was set to 0.00. Measurements were taken using ImageJ [27]. For statistical calculations, data of the two females were averaged for each testing container. The differences in [RCG t_max_−RCG t_0_]-values (with RCG t_max_ being the RCG_max_ between t_0_ and t_60_) between courtship water and aged tap water were tested with the non-parametric two-tailed Mann-Whitney U test [28] using SPSS [29]. Twenty females were used in the test, and each female was used twice: one time for the CW test and once for the negative control. Ten females were first subjected to the CW test, the other 10 females were first subjected to the negative control.

### Courtship water sampling for behavioral tests

A male and a female were placed in a plastic container (25x16x14 cm) filled with 2L of aged tap water. Once in amplexus, couples were monitored for courtship behavior and the amount of time that a male released one forelimb and imposed his protruded cloaca onto the female’s nose was measured. We termed this behavior *cloacal imposition*, and interpreted it as the moment of pheromone transfer [25]. We only retained water of courting couples if at least 30 minutes of male cloacal imposition occurred and if the female responded with cloacal gaping and a pinwheel movement. The couple was manually separated before spermatophore drop and animals were returned to their housing tanks. The successfully obtained courtship waters were mixed and equally divided over the test containers for the cloacal gaping tests.

## Results and Discussion

### Cloacal gaping precedes pinwheel behavior

**Fig 1:**
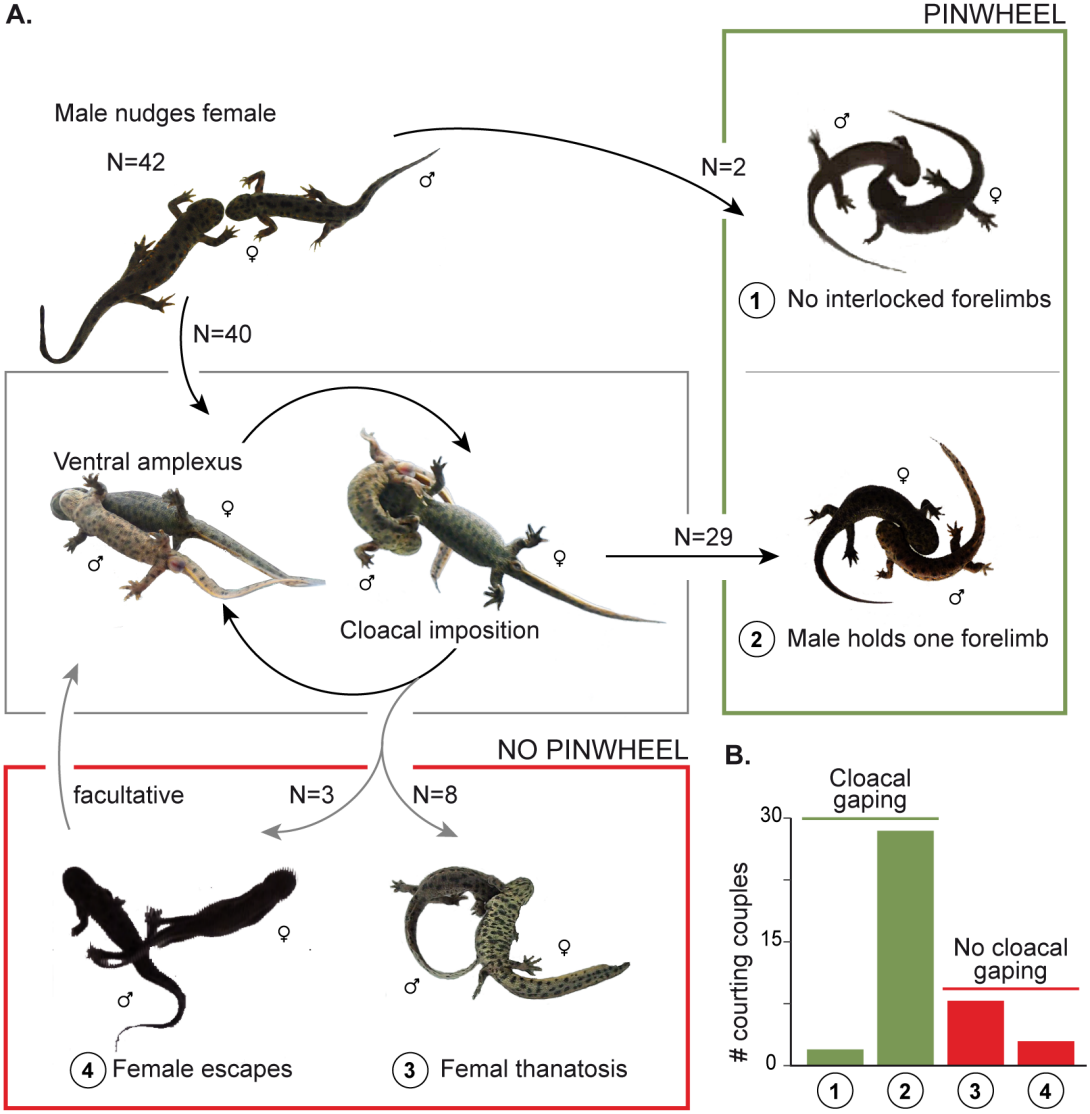
Courtship behavior of *Pleurodeles waltl*. **A)** Observed outcomes. The first outcome results from a female that is interested from the first male approach onwards. After male nudging, the couple immediately continues into pinwheel behavior without contact (i.e., skipping amplexus). The second and most common outcome is obtained in three steps, in the following order: nudging (first approach; FA), ventral amplexus (VA) altered with cloacal imposition (CI), and pinwheel behavior (PW) with one interlocked forelimb. In outcomes 1 and 2, female cloacal gaping (FCG) was always evident (indicated in green). In the third and fourth outcomes, female thanatosis and female struggle both lead to abortion of courtship, and no female cloacal gaping occurred (indicated in red). The number of times that each outcome occurred (out of 42 observations) is added. **B)** Graph of courtship observations. Number of times that courtship outcomes occurred during the observation of 42 couples.

Successful insemination in *Pleurodeles waltl* always results from pinwheel behavior (PW), i.e. the couple going into a circular movement that leads to spermatophore deposition by the male, and subsequent uptake by the female cloaca [3,24,25,30]. Our observations of the courtship behavior of 42 couples showed that, after the first contact in which they nudge the snout and/or body flank of the female, males could reach such pinwheel behavior in two ways (Fig 1A). First, when the female immediately responded positively to the nudging male (2/42 observations), the male was triggered to pivot around the female, while she followed him in pinwheel behavior without interlocked forelimbs (Fig 1A, outcome 1; S1 Video). Insemination without a preceding amplexus has rarely been described in the genus *Pleurodeles* [31], and seems to be most successful when a female is already highly receptive. Male courtship behavior that is dependent on the female’s responsiveness (facultative amplexus) has also been found in the red-spotted newt (*Notophthalmus viridescens*) [30,32]. As far as known, other salamandrid species never (most Modern Eurasian newts, *Echinotriton*, some *Tylototriton* species, *Salamandrina*) or always (*Taricha*, *Calotriton*, *Euproctus*, some *Tylototriton* species, *Salamandra*, *Mertensiella*) display a type of amplexus behavior during courtship [3,33].

Most often (40/42 observations), the female did not directly engage in pinwheel behavior and showed no interest, in which cases the male swam under the female to clasp her forelimbs in a ventral amplexus (VA) with his arms in a 90° angle [24,25] (Fig 1A). Amplexus in newts enables the male to monopolize a female, but also facilitates applying his courtship pheromone glands to the restrained female [30]. Once in ventral amplexus, the male’s cloaca became swollen and protruded, and he started alternating his ventral amplexus position with cloacal impositions (CI; thereby releasing one forelimb—in 92.5% of the cases the left one; Fig 1A; S2 Video). Glands in the cloacal region are a known source of pheromones in multiple salamandrid species [2,4,6,30,34–36], and these cloacal impositions are thus the obvious moments where the male applies his pheromones to the female’s nose [30]. After a period of alternating ventral amplexus with cloacal imposition, 29 females showed a first sign of receptivity by cloacal gaping (i.e. opening their cloaca; FCG), which finally (more than four hours later) resulted in coordinated pinwheel behavior, with the male still holding one of the female's forelimbs (Fig 1A, outcome 2; S3 Video).

It is generally believed that an amplected female amphibian does not have much choice other than to reproduce with the clasping male [4,23,37,38]. However, 11 amplected females avoided the stage of pinwheel behavior by either thanatosis (i.e., feigning death) or escaping from amplexus. Eight females avoided spermatophore pick-up by thanatosis (Fig 1A, outcome 3; S4 Video), during which males were not able to properly reach the female’s nose with their cloacal glands. Thanatosis was shown to be an effective strategy that invariably terminated the amplexus. To our knowledge, thanatosis in amphibians is so far only known as a defense mechanism against predators [4,39], but has not been observed in a sexual context. In fact, throughout the entire Animal Kingdom, this behavior is exceptional during courtship and is known only in spiders and insects. In spiders, thanatosis is performed by the male during courtship to avoid being eaten by the female [40], while in the fly *Efferia varipes*, it is similarly used as a strategy to get rid of harassing mates [41]. Finally, three females struggled themselves out of the male’s grip at the moment the male started his first cloacal imposition (Fig 1A, outcome 4). In each case, the male responded by tail-fanning, likely to transfer his pheromones in an alternative way. This behavior in itself never resulted in cloacal gaping, but once resulted in a new successful ventral amplexus with subsequent pinwheel behavior. Tail-fanning has already been described as a prelude to, and once even as a replacement of amplexus in *Pleurodeles waltl* [25,31], but we only observed it as this secondary, rather unsuccessful tactic.

Importantly, our observations showed that pinwheel behavior (whether obtained directly or after ventral amplexus) was always preceded by cloacal gaping, i.e., the female opening her cloaca as a prelude of spermatophore pick-up (Fig 1B). Our timing of complete courtship sequences indicated that this reaction occurred 12 to 51 minutes (N = 11; mean = 31 min; standard deviation = 14 min) after the first male cloacal imposition, while the transition from the first cloacal imposition to pinwheel behavior (for spermatophore pick-up; see below) took much longer, from 149 up to 310 minutes (N = 11; mean = 227 min; standard deviation = 55 min) (Table 1). In contrast, none of the females that escaped or feigned death showed cloacal gaping, indicating that this modification is a reliable indicator for a female being ready for spermatophore pick-up. Since most of the successful pinwheel behaviors were induced by cloacal imposition, and thus use of pheromones, we next investigated whether this link could be used for constructing a behavioral test with control over the presence of courtship pheromones in the absence of a male.

**Table 1:** Timing of courtship behaviors in *Pleurodeles waltl* couples.

Nr.	FA	VA	CI	FCG	PW	CI → FCG	CI → PW
**1**	0	1	8	53	276	45	268
**2**	0	9	24	68	189	44	165
**3**	4	21	26	65	336	39	310
**4**	0	2	7	23	243	16	236
**5**	0	2	5	17	262	12	257
**6**	5	6	14	50	198	36	184
**7**	1	2	12	31	214	19	202
**8**	3	13	19	70	168	51	149
**9**	0	3	5	26	178	21	173
**10**	0	0	8	27	284	19	276
**11**	4	4	10	52	288	42	278
**Mean**	/	/	13	44	240	31	227
**Stand Dev**	/	/	7	20	54	14	55

The values represent the time (in minutes) that specific courtship behaviors took place (N = 11). The time is measured from the moment that the animals are together in the tank. FA: first approach (male nudges the female), VA: start of ventral amplexus, CI: moment of the first male cloacal imposition (pheromone transfer), FCG: start of female cloacal gaping, PW: start of pinwheel behavior, CI → FCG: time between the first cloacal imposition and female cloacal gaping, CI → PW: time between the first cloacal imposition and pinwheel behavior.

### Female cloacal gaping as a bioassay for demonstrating courtship pheromone use in newts

**Fig 2:**
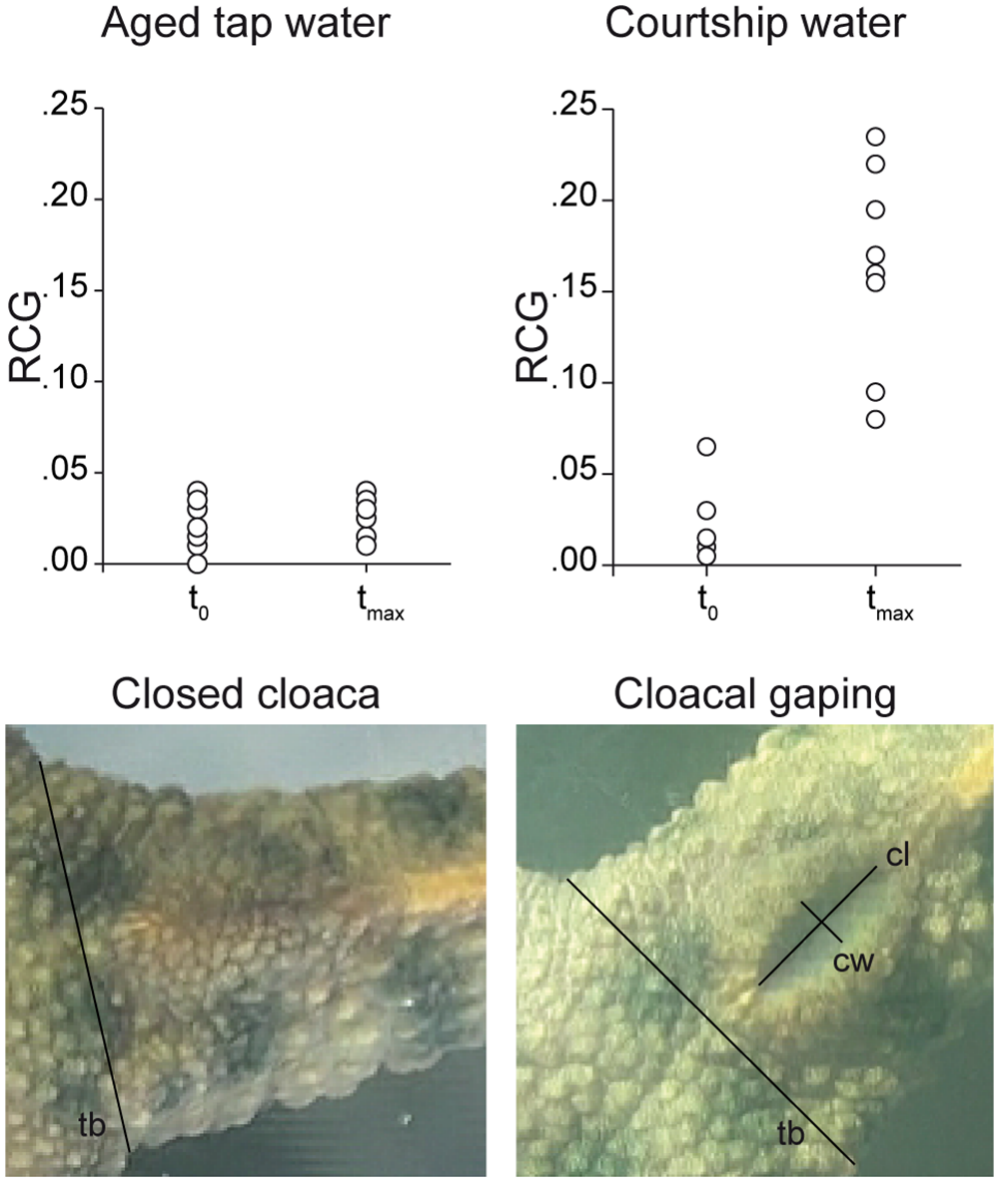
Cloacal gaping in females. The graphs represent female Relative Cloacal Gaping (RCG) at t_0_ and t_max_ in courtship water and aged tap water (negative control). RCG was measured as the cloacal width (cw) divided by the tail base width (tb). The width of the cloaca was measured orthogonal to the center of the cloacal length (cl). The tail base was measured at the hind limb initiation region. If the cloaca was closed, RCG was set to 0.00.

To check whether female cloacal gaping could be evoked by male pheromones, we monitored the cloaca of females in courtship water (water in which a male has been courting, thus containing pheromones; see Methods) for 60 minutes. When females (N = 20) were exposed to courtship water, their cloaca observably changed from a closed to a more open state (Fig 2; Table 2). Although not every cloaca was in a completely closed state at the start of the test, gaping was enhanced in all of them during exposition to courtship water. Overall, the difference in relative cloacal gaping between the maximum (at t_max_) and onset of the test (at t_0_) was significantly larger than that observed in the negative control (*U* = 0.00, *N*_*1*_ = *N*_*2*_ = 10, *P* < 0.001). These results suggest that pheromones in courtship water induce cloacal gaping in females, even in the absence of males. Cloacal gaping as a female response to males is also known in several snake species, where males perform caudocephalic waving (i.e. muscular contractions from cloaca to head) towards females [42,43], and the female responds by lifting her tail and gaping her cloaca [44,45]. In snakes and salamanders, a gaped female cloaca results from successful male courtship, and serves as the prelude for either inserting the hemipenis [46] or picking up the spermatophore, respectively.

**Table 2:** Relative Cloacal Gaping (RCG) in females of *Pleurodeles waltl*.

Courtship water				Negative control			
	RCG at t_0_	t_max_ (min)	RCG at t_max_		RCG at t_0_	t_max_ (min)	RCG at t_max_
F1	0.01	30	0.21	F1	0.01	25	0.03
F2	0.02	25	0.10	F2	0.03	10	0.03
F3	0.02	20	0.13	F3	0.03	0	0.03
F4	0.01	30	0.03	F4	0.04	0	0.04
F5	0.12	10	0.30	F5	0.00	15	0.01
F6	0.01	25	0.02	F6	0.00	25	0.01
F7	0.01	25	0.19	F7	0.03	0	0.03
F8	0.00	35	0.28	F8	0.03	0	0.03
F9	0.00	40	0.24	F9	0.03	0	0.03
F10	0.01	10	0.15	F10	0.00	10	0.04
F11	0.03	20	0.23	F11	0.03	0	0.03
F12	0.03	45	0.21	F12	0.01	15	0.02
F13	0.02	20	0.25	F13	0.02	0	0.02
F14	0.01	35	0.09	F14	0.00	20	0.01
F15	0.02	40	0.14	F15	0.03	0	0.03
F16	0.00	30	0.02	F16	0.01	45	0.02
F17	0.00	60	0.16	F17	0.00	/	0.00
F18	0.03	15	0.03	F18	0.02	15	0.02
F19	0.01	20	0.01	F19	0.04	0	0.04
F20	0.02	20	0.15	F20	0.04	0	0.04

Relative Cloacal Gaping (RCG) in females (F) at the start of the bioassay (t_0_) and at the time of maximum cloacal gaping (t_max_). Females were coupled per testing container. Female numbers are associated with the test order and not with individuals.

In internally fertilizing salamanders, males do not make use of a copulatory organ, but instead deposit a spermatophore in the environment that females take up with their cloaca. Opening of the cloaca is thus a logical prelude to insemination. Although cloacal gaping does not necessarily have to be dependent on pheromone use, our observations during a two-female test with *Lissotriton helveticus* in a previous study [6] indicate that cloacal modification—in the absence of a male but in the presence of male pheromones—is also evident in this species (S5 Video). We therefore hypothesize that our bioassay, with small modifications, will be useful for identifying pheromones in a broad range of internally fertilizing species with different courtship strategies.

## Conclusion

It is generally acknowledged that many salamanders make use of courtship pheromones to persuade females into reproduction. These courtship pheromones are species-specific, are secreted by various glands, and are delivered by several behavioral tactics. However, studies that have effectively characterized such molecules until now have been limited [47], partly due to the difficulties of designing appropriate behavioral tests [48]. A behavioral test is often specifically designed and optimized for pheromone identification in a single species and its closest relatives. However, when aiming at understanding courtship pheromone evolution in salamanders, the availability of an assay that involves a comparable response across multiple species, genera or families, would be a strong asset. This study describes a behavioral test for identifying courtship pheromones that opens perspectives for use in multiple species of salamanders.

## Supporting Information

S1 Video Pinwheel behavior without interlocked forelimbs. The male nudges the female during first contact. The female (recognized by her thicker belly) immediately responds with pinwheel behavior, so the couple circles around without interlocked forelimbs until sperm transfer takes place. DOI: http://dx.doi.org/10.6084/m9.figshare.1612191.
(DOC)

S2 Video Cloacal imposition. When a male (below) is in ventral amplexus with a female (above), his cloaca protrudes and the male starts alternating this behavior with cloacal impositions: he releases one forelimb, and rotates around the female’s head to impose his cloaca on the female’s nose. After a while (Mean value of 31 minutes; calculated from the data in Table 1), the female responds by opening her cloaca, but other observable responses are not performed until much later. DOI: http://dx.doi.org/10.6084/m9.figshare.1612192.
(DOC)

S3 Video Pinwheel behavior with interlocked forelimbs. After alternating ventral amplexus with cloacal imposition, the couple initiates pinwheel behavior, while one of the female’s forelimbs is still held by the male’s forelimb. This courtship behavior usually ends in a successful sperm transfer. DOI: http://dx.doi.org/10.6084/m9.figshare.1612194.
(DOC)

S4 Video Female thanatosis. One of the strategies that females use to escape from an amplexus is thanatosis. The female feigns death until the male gives up. DOI: http://dx.doi.org/10.6084/m9.figshare.1612195.
(DOC)

S5 Video Cloacal gaping in *Lissotriton helveticus*. A two-female test with two palmate newts in courtship water illustrates that the female showing following behavior under influence of SPF courtship pheromones [6] has a clearly extended cloaca. DOI: http://dx.doi.org/10.6084/m9.figshare.1612196.
(DOC)
