# Supplementary material for: Male Courtship Pheromones Induce Cloacal Gaping in Female Newts (Salamandridae)
Source: PLoS One. 2016 Jan 15;11(1):e0144985. doi: 10.1371/journal.pone.0144985 (PMC4714853; doi:10.1371/journal.pone.0144985)
Supplement: S1 Video — The male nudges the female during first contact. The female (recognized by her thicker belly) immediately responds with pinwheel behavior, so the couple circles around without interlocked forelimbs until sperm transfer takes place. DOI: http://dx.doi.org/10.6084/m9.figshare.1612191. (DOC) [file pone.0144985.s001.doc]

**S1 Video: Pinwheel behavior without interlocked forelimbs.** The male nudges the female during first contact. The female (recognized by her thicker belly) immediately responds with pinwheel behavior, so the couple circles around without interlocked forelimbs until sperm transfer takes place.

DOI: [http://dx.doi.org/10.6084/m9.figshare.1612191](http://dx.doi.org/10.6084/m9.figshare.1612191)
