# Supplementary material for: Male Courtship Pheromones Induce Cloacal Gaping in Female Newts (Salamandridae)
Source: PLoS One. 2016 Jan 15;11(1):e0144985. doi: 10.1371/journal.pone.0144985 (PMC4714853; doi:10.1371/journal.pone.0144985)
Supplement: S2 Video — When a male (below) is in ventral amplexus with a female (above), his cloaca protrudes and the male starts alternating this behavior with cloacal impositions: he releases one forelimb, and rotates around the female’s head to impose his cloaca on the female’s nose. After a while (Mean value of 31 minutes; calculated from the data in Table 1), the female responds by opening her cloaca, but other observable responses are not performed until much later. DOI: http://dx.doi.org/10.6084/m9.figshare.1612192. (DOC) [file pone.0144985.s002.doc]

**S2 Video: Cloacal imposition**. When a male (below) is in ventral amplexus with a female (above), his cloaca protrudes and the male starts alternating this behavior with cloacal impositions: he releases one forelimb, and rotates around the female’s head to impose his cloaca on the female’s nose. After a while (Mean value of 31 minutes; calculated from the data in Table 1), the female responds by opening her cloaca, but other observable responses are not performed until much later.

DOI: [http://dx.doi.org/10.6084/m9.figshare.1612192](http://dx.doi.org/10.6084/m9.figshare.1612192)
