# Supplementary material for: Male Courtship Pheromones Induce Cloacal Gaping in Female Newts (Salamandridae)
Source: PLoS One. 2016 Jan 15;11(1):e0144985. doi: 10.1371/journal.pone.0144985 (PMC4714853; doi:10.1371/journal.pone.0144985)
Supplement: S3 Video — After alternating ventral amplexus with cloacal imposition, the couple initiates pinwheel behavior, while one of the female’s forelimbs is still held by the male’s forelimb. This courtship behavior usually ends in a successful sperm transfer. DOI: http://dx.doi.org/10.6084/m9.figshare.1612194. (DOC) [file pone.0144985.s003.doc]

**S3 Video: Pinwheel behavior with interlocked forelimbs.** After alternating ventral amplexus with cloacal imposition, the couple initiates pinwheel behavior, while one of the female’s forelimbs is still held by the male’s forelimb. This courtship behavior usually ends in a successful sperm transfer*.*

DOI: [http://dx.doi.org/10.6084/m9.figshare.1612194](http://dx.doi.org/10.6084/m9.figshare.1612194)
