# Supplementary material for: Male Courtship Pheromones Induce Cloacal Gaping in Female Newts (Salamandridae)
Source: PLoS One. 2016 Jan 15;11(1):e0144985. doi: 10.1371/journal.pone.0144985 (PMC4714853; doi:10.1371/journal.pone.0144985)
Supplement: S4 Video — One of the strategies that females use to escape from an amplexus is thanatosis. The female feigns death until the male gives up. DOI: http://dx.doi.org/10.6084/m9.figshare.1612195. (DOC) [file pone.0144985.s004.doc]

**S4 Video: Female thanatosis.** One of the strategies that females use to escape from an amplexus is thanatosis. The female feigns death until the male gives up.

DOI: [http://dx.doi.org/10.6084/m9.figshare.1612195](http://dx.doi.org/10.6084/m9.figshare.1612195)
