# Supplementary material for: Male Courtship Pheromones Induce Cloacal Gaping in Female Newts (Salamandridae)
Source: PLoS One. 2016 Jan 15;11(1):e0144985. doi: 10.1371/journal.pone.0144985 (PMC4714853; doi:10.1371/journal.pone.0144985)
Supplement: S5 Video — A two-female test with two palmate newts in courtship water illustrates that the female showing following behavior under influence of SPF courtship pheromones [6] has a clearly extended cloaca. DOI: http://dx.doi.org/10.6084/m9.figshare.1612196. (DOC) [file pone.0144985.s005.doc]

**S5 Video: Cloacal gaping in *Lissotriton helveticus*.** A two-female test with two palmate newts in courtship water illustrates that the female showing following behavior under influence of SPF courtship pheromones [6] has a clearly extended cloaca.

DOI: [http://dx.doi.org/10.6084/m9.figshare.1612196](http://dx.doi.org/10.6084/m9.figshare.1612196)
